# Birth weight of term born offspring in relation to long-term maternal cardiovascular morbidity and mortality

**DOI:** 10.1007/s10654-025-01355-1

**Published:** 2026-02-21

**Authors:** Pauline Kromann Reim, Line Engelbrechtsen, Lise Geisler Bjerregaard, Louise Kelstrup, Erik Lykke Mortensen, Thorkild I. A. Sørensen, Torben Hansen

**Affiliations:** 1 Novo Nordisk Foundation Center for Basic Metabolic Research, University of Copenhagen, Copenhagen, Denmark; 2 Department of Gynaecology and Obstetrics, Herlev University Hospital, Herlev, Denmark; 3 Center for Clinical Research and Prevention, Copenhagen University Hospital – Bispebjerg and Frederiksberg, Copenhagen, Denmark; 4 Department of Public Health, University of Copenhagen, Copenhagen, Denmark; 5 Center for Healthy Aging, University of Copenhagen, Copenhagen, Denmark; 6 Center for Childhood Health, University of Copenhagen, Copenhagen, Denmark; 7 Department of Clinical Medicine, University of Copenhagen, Copenhagen, Denmark

**Keywords:** Birth weight, Pregnancy, Maternal health, Cardiovascular disease

## Abstract

Previous studies have identified associations between offspring birth weight (BW) and future maternal cardiovascular health, but many studies lack sufficient adjustment for confounders such as pregnancy complications, gestational age, maternal body mass index (BMI), and smoking. This study aimed to assess whether the association between term offspring BW and maternal cardiovascular risk is independent of these factors. We used data from the Copenhagen Perinatal Cohort (1959–1961). After excluding women with diabetes, preeclampsia, preterm births, and twin pregnancies, 5,766 women remained. Cardiovascular outcomes were obtained from Danish national registries with up to 45 years of follow-up. Hazard ratios (HR) with 95% confidence intervals (CI) were estimated using Cox regression, adjusting for smoking during pregnancy, maternal BMI, hypertension, and gestational age at birth. Additional analyses were conducted in populations stratified by smoking status and adjusted for cigarette consumption per day. Offspring BW below 3000 grams (g) was associated with higher maternal cardiovascular mortality (< 2500 g: 2.16 [1.54–3.03], 2500–2999 g: 1.41 [1.09–1.81]) compared to the reference category (offspring BW 3500–3999 g). The risks of maternal major cardiovascular events and stroke were higher when offspring BW was below 2500 g (1.35 [1.01–1.79], 1.51 [1.09–2.08]) and lower if offspring BW was above 3999 (0.78 [95% CI 0.61–0.99], 0.78 [0.59–1.03]) compared to the reference. No associations were found with ischemic heart disease. Associations persisted after stratification by smoking. Lower BW of term offspring from uncomplicated, singleton pregnancies is inversely associated with long-term maternal cardiovascular risk, independent of smoking during pregnancy.

**Supplementary Information:**

The online version contains supplementary material available at 10.1007/s10654-025-01355-1.

## Introduction

The accommodation of the maternal body to the physiological demands and cardiovascular adaptations of pregnancy can enhance or expose underlying disease risk, and has been suggested to offer an early-life stress-test predicting future maternal health [1, 2]. As such, pregnancy may provide a window of opportunity to postpone or prevent development of manifest disease [3–5]. Being the endpoint of intrauterine growth, offspring birth weight (BW) may serve as an indicator of the extent to which these maternal adaptations have succeeded and hereby provide valuable information about the future health of the mother. Observational studies have revealed an inverse association between offspring BW and subsequent maternal cardiovascular disease (CVD) [6–8] and mortality [9–11]. However, the underlying mechanisms behind these associations remain unclear and are likely affected by pregnancy characteristics and shaped by a combination of genetic and environmental factors [12]. Most previous studies evaluating the association between offspring BW and maternal health and disease are limited by factors possibly introducing significant bias to the results: either lack of reliable information about offspring BW [13], gestational age (GA) at birth [6, 9] or insufficient adjustment for potential confounding factors such as maternal medical conditions during pregnancy (including pregnancy complications) [7, 8, 10], and smoking during pregnancy [6–10].

The fetus has the most substantial weight gain during the third trimester with a fetal weight velocity peak around week 35 [14]. If GA is not accounted for, premature offspring may be categorized as growth restricted even when they are appropriate in size or large for their GA [7]. In addition, fetal growth as well as future maternal health [2] is known to be affected by maternal BMI [15], preeclampsia [16], hypertension [17], and diabetes [18]. Consequently, the associations observed between offspring BW and maternal health outcomes could be due to other pregnancy complications known to affect both exposure and outcome and not fetal growth restriction per se, resulting in inflation of the strength of the association [19].

Lastly, smoking during pregnancy is a potential confounder in the association, as it is known to strongly associate with both lower offspring BW [20, 21] and increased long-term morbidity and mortality in the mother [22]. In addition, smoking during pregnancy may modify the association between offspring BW and maternal CVD risk, potentially reflecting different underlying mechanisms of fetal growth restriction and maternal vascular dysfunction in smokers versus non-smokers. Yet, few previous studies have included data on smoking during pregnancy [11, 23]. One study excluded smoking from their mortality analyses because the data were available for only about 25% of participants and suggested that unmeasured confounding could explain the observed associations [11]. Moreover, none of these studies examined the associations specifically among women who did not smoke during pregnancy or evaluated whether smoking modified the relationship between offspring BW and maternal long-term health.

Accurately assessing the association between offspring BW and long-term maternal health requires careful consideration of the factors that may partially drive the relationship. If offspring BW is indeed a marker of future maternal CVD risk, this would suggest that the mechanisms contributing to maternal CVD also influence fetal growth.

We hypothesized that offspring BW is inversely associated with maternal all-cause and cardiovascular mortality and morbidity, even after adjusting for known confounding factors, suggesting that BW may represent an independent marker of future maternal health. This hypothesis was investigated in a pregnancy cohort with detailed maternal health information collected before and during pregnancy, and linked to national registries, enabling up to 45 years of follow-up. The study also assessed whether smoking during pregnancy modified the observed associations.

## Materials and methods

### Population

This study used data from the Copenhagen Perinatal Cohort (CPC [24, 25]) consisting of 8949 women from the Capital Region, Denmark, included during pregnancy and giving birth at Copenhagen University Hospital, Rigshospitalet, Denmark, from September 1959 to December 1961. Information on maternal age at inclusion (years), marital status (married yes/no), employment status (employed in pregnancy yes/no), pre-pregnancy body-mass-index (BMI, weight (kg)/height (m)^2^), twin pregnancy (yes/no), pre-eclampsia (yes/no), eclampsia (yes/no), diabetes (yes/no), hypertension (yes/no), and smoking during pregnancy was available. Information on smoking during pregnancy was registered in the third trimester as either yes or no and as cigarette consumption per day divided into five categories: 0 cigarettes, < 3 cigarettes, 3–10 cigarettes, 11–20 cigarettes and > 20 cigarettes. Information on diabetes did not specify the type of disease (type 1 or 2 diabetes or gestational diabetes). It was not recorded whether hypertension was present before pregnancy or not. GA was calculated based on the number of days between the date of the first day in the woman’s last menstrual period and the date of delivery divided by seven to obtain GA in weeks.

The following women were excluded from the final analyses: Women who died during pregnancy or delivery (n = 7), women with missing information on GA (n = 1564), with invalid information on GA (either negative values or values > 50 weeks) (n = 73), with missing information on offspring BW (n = 14), with pre-eclampsia or eclampsia (n = 284), women with diabetes (n = 75), twin births (n = 110), premature births (defined as GA < 37 weeks) (n = 825), still births (n = 1), women with no available Central Person Register number (CPR-number) (n = 17), and with missing information on smoking during pregnancy (n = 115). For the mortality analyses we excluded women who either died, emigrated or were lost-to-follow-up before follow-up start (n = 98), and for the morbidity analyses we also excluded women who either died, emigrated or where lost-to-follow-up before follow-up start (n = 176). The final population consisted of 5766 women for the mortality analyses and 5688 for the morbidity analyses.

**Fig. 1:**
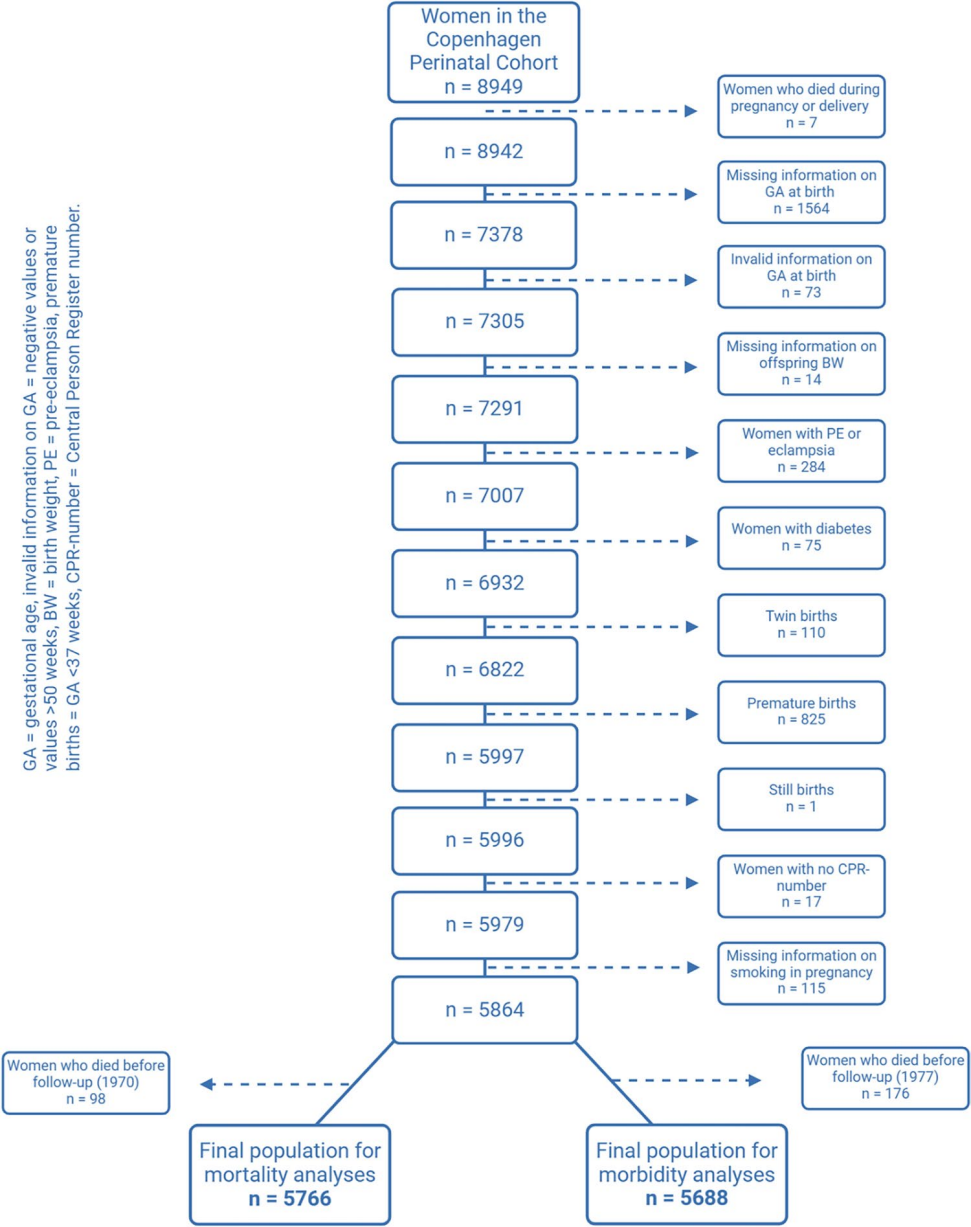
Flow chart of population exclusions

An overview of the exclusion flow is provided in Fig. 1.

### Exposure indicator

The exposure was indicated by BW of offspring delivered at term or post-term (GA >  = 37 weeks). Offspring BW was divided into a categorical variable of 5 five categories: < 2500, 2500–2999, 3000–3499, 3500–3999, and > 3999 g. Offspring BW of 3500–3999 was defined as the reference category.

### Outcome

To obtain information on mortality and cause of death, the women in the final study population were linked to the Danish Cause of Death Registry (DCDR) [26] by their personal identification number from the Danish Central Person Register (CPR). Variables extracted from the register per December 31st, 2015, included information on date and cause of death based on the International Classification of Diseases (ICD). We evaluated all-cause mortality and cardiovascular mortality, defined as cause of death by either hypertension (I10-11), ischemic heart disease (IHD) (I20-21 and I24-25), stroke (ischemic and hemorrhagic) (I60-69), and diseases in the arteries, arterioles, and capillaries (I70-79). In addition, we evaluated smoking-related mortality, defined as cause of death by smoking-related cancers (cancers of the oral cavity (C00-14), esophagus (C15) and airways (C33-34)), chronic obstructive pulmonary disease, and asthma (J41-47).

Information on cardiovascular morbidity was obtained from the Danish National Patient Register (DNPR) [27] and included information on cardiovascular diagnoses received upon or during an admission to a hospital unit in Denmark. Diagnoses were based on the ICD8 (1977–1993) and ICD10 (1994–2017). Diagnoses before January 1st, 1977, were not available for analysis. Cardiovascular diagnoses included: ischemic heart disease (IHD) (I20-21 and I24-25), hypertension (I10-11), stroke (ischemic and hemorrhagic) (I60-66), and a combined outcome of major cardiovascular events (MACE) defined as the diagnosis of either acute myocardial infarction (I21), stroke (I60-69), cardiac arrest, or sudden cardiac death (I46). The dates of first-time diagnoses for each disease entity were included in the analyses. The analyses were conducted separately for each diagnosis (IHD, hypertension, stroke, MACE) allowing the individuals to be part of the at-risk population for all the evaluated CVD outcomes.

Variables were extracted in August 2017 and were available from both registers until December 31st, 2015. Follow-up started on January 1st, 1970, for DCDR data and January 1st, 1977, for DNPR data providing follow-up periods of up to 45 years and 38 years respectively. Follow-up ended on the date of death, emigration, loss to follow-up (changed CPR-number, disappeared, no registered residence in Denmark), diagnosis of the CVD in question, or December 31st, 2015, whichever came first.

### Ethics

According to Danish law (§14, part 2 of the Danish Act on Ethical Conduct of Health Research), this study did not require approval from a scientific ethics committee, as it was based on information retrieved from health registries. Women were enrolled in the Copenhagen Perinatal Cohort during routine admission procedures at Copenhagen University Hospital between 1959 and 1961, and most data were obtained through standard clinical care. At that time, formal consent procedures were not in place, but participants were thoroughly informed about the investigations and assured of confidentiality.

### Statistics

All analyses were carried out using R version 4.4.2 (http://www.r-project.orgwww.r-project.org).

Linearity in the association between exposure (offspring BW) and outcome (maternal all-cause mortality) was assessed using splines and revealed a non-linear relationship (Linear vs. Spline Model plot is available in Supplementary Fig. S1a and S1b).

Offspring BW according to each category of number of cigarette consumption per day in pregnancy was assessed in the total population and illustrated in a boxplot. The difference between groups was assessed using ANOVA and the post-hoc analyses tool, Tukey’s Honest Significant Difference (HSD) test, was performed to assess pairwise differences while adjusting for multiple comparisons.

Crude survival was illustrated in Kaplan–Meier Plots and Log-Rank tests were used to conduct pairwise comparisons of survival according to offspring BW. All-cause mortality, cardiovascular mortality, smoking-related mortality, and cardiovascular morbidity were estimated using Cox proportional hazard models providing hazard ratios (HRs) and 95% confidence intervals (CI) in relation to offspring BW category. Age was used as the underlying time variable. The analyses were adjusted for maternal age at inclusion, pre-pregnancy BMI, sex of the offspring, GA, marital status, employment status, hypertension and smoking status in pregnancy (smoking yes/no). The proportional hazards assumption was assessed for each covariate in the models using Schoenfeld residuals.

Likelihood Ratio Tests were performed to evaluate the potential confounding effect of smoking during pregnancy (yes/no) on the exposure (offspring BW) and the outcomes (maternal mortality and morbidity). A reduced model, not including smoking as a covariate, and a full model, including smoking, were compared.

To explore the possible modifying effect of smoking during pregnancy, all analyses were carried out in the total population as well as in populations stratified by smoking during pregnancy (yes/no). To account for the potential dose-dependent effects of smoking on both fetal growth [21, 28] and maternal CVD risk, the analyses of the population of women who smoked during pregnancy were adjusted for cigarette consumption per day in pregnancy. Likelihood ratio tests were used to compare a model with smoking as a covariate to a model with offspring BW and smoking as an interaction term.

## Results

A total of 5766 women were included in the mortality analyses and 5688 in the cardiovascular morbidity analyses (Fig. 1).

The women in the total population (n = 5766) were on average 25.3 years old (SD = 6.50) at inclusion and had a pre-pregnancy BMI of 21.75 (SD = 2.81). The mean offspring BW was 3320 g (SD = 493), mean GA was 40.5 weeks (SD = 1.88), and 49.6% of the offspring were females. Table 1 provides a full overview of population characteristics. More than half of the women smoked during pregnancy (51.8%). The women in the smoking and non-smoking populations were similar in age, pre-pregnancy BMI, female/male offspring ratio, and duration of pregnancy (GA at birth). The mean offspring BW was higher for women who did not smoke during pregnancy than for women who did (3430 g vs. 3220 g) and there was a higher proportion of hypertension during pregnancy in the population of women who did not smoke during pregnancy (25% vs. 15%). Characteristics of the sub-populations are available in Supplementary table S1.

**Table 1:** Population characteristics

Population Characteristics		
n		5766
Offspring Birth Weight (g), mean (SD)		3320 (0.5)
Offspring Birth Length (cm), mean (SD)		51 (2)
Gestational Age at Delivery (weeks), mean (SD)		40.5 (1.88)
Sex of Offspring, n (%)	F	2858 (49.6)
	M	2908 (50.4)
Caesarean Section, n (%)	No	5396 (93.6)
	Yes	370 (6.4)
Maternal Age (years), mean (SD)		25 (6.5)
Pre-pregnancy BMI, mean (SD)		21.8 (2.8)
Marital Status, n (%)	Married	3494 (60.6)
	Not married	2272 (39.4)
Employed in Pregnancy, n (%)	No	2013 (34.9)
	Yes	3753 (65.1)
Smoking During Pregnancy, n (%)	No	2777 (48.2)
	Yes	2989 (51.8)
Hypertension in Pregnancy, n (%)	No	4594 (80.5)
	Yes	1115 (19.5)
Offspring Birth Weight Category, n (%)	< 2500	298 (5.2)
	2500–2999	965 (16.7)
	3000–3499	2335 (40.5)
	3500–3999	1637 (28.4)
	> 3999	531 (9.2)

Offspring BW was significantly higher among women who smoked fewer than three cigarettes per day during pregnancy compared to those who smoked more than three per day. Beyond this distinction, no significant differences in offspring BW were observed with increasing levels of maternal cigarette consumption per day in pregnancy (Supplementary Fig. S2 and table S2).

No major violations of the proportional hazard’s assumption were observed for most covariates (p-values > 0.05, Supplementary Table S3); however, smoking during pregnancy, maternal age at birth, and maternal pre-pregnancy BMI showed significant evidence of non-proportionality (p = 0.00044, 0.00016, and 0.00004, respectively). Visual inspection of the Schoenfeld residuals suggested only minor deviations, with no substantial time-dependent trends for any of the covariates (plots in Supplementary Fig. 3a-c). Therefore, the covariates were retained in the model without time-varying adjustment.

The Likelihood Ratio Test revealed that smoking during pregnancy was a significant confounding factor in the associations between offspring BW and all outcomes evaluated in the study, except for hypertension. Only for the associations between offspring BW and risk of MACE the model with the interaction term between smoking and offspring BW appeared to be slightly superior (p = 0.032) compared to the model with smoking as a covariate (Supplementary table S4a + b).

### Crude survival

At the end of follow-up, 3128 out of 5766 (~ 54%) women were deceased and 93 were censored due to either emigration or loss to follow-up before the event of death occurred. In the population of women who smoked during pregnancy ~ 64% (1911/2989) were deceased at end of follow-up, and in the population who did not smoke during pregnancy ~ 44% (1217/2777) were deceased. In the total population and the population of women who smoked during pregnancy, crude survival analysis showed a lower survival rate in women giving birth to offspring with a BW below 3000 g compared to women giving birth to offspring with a BW above 3000 g (Supplementary Fig. S4 and S5). The lowest survival rate was observed in women giving birth to offspring with BW of < 2500 g in both populations (Supplementary Fig. S4 and S5). In the population of women who did not smoke during pregnancy, crude survival analysis showed a lower survival rate in women giving birth to offspring with a BW of < 2500 g compared with women giving birth to offspring between 2500–3999 g (Supplementary Fig. S6).

### Mortality

In the total population, we found a higher all-cause mortality and cardiovascular mortality in women giving birth to offspring below 3000 g compared to women giving birth to offspring with a BW in the reference category (3500–3999 g) (Table 2). In women giving birth to offspring with a BW of < 2500 g and 2500–2999 g, the HRs for all-cause mortality were 1.71 (95% CI 1.45–2.00) and 1.23 (95% CI 1.20–1.38), and for cardiovascular mortality 2.16 (95% CI 1.54–3.03) and 1.41 (95% CI 1.09–1.81), respectively (Table 2). For cardiovascular mortality there was a tendency towards a lower mortality in women giving birth to offspring above 3999 g, though this was non-significant (HR 0.87 [95% CI 0.62–1.20]). Smoking-related mortality was higher in women giving birth to offspring in all BW categories below 3500 g compared with the reference category (Table 2).

**Table 2:** Mortality in women in relation to offspring birth weight

Total population of women*																					
	All-cause mortality (n events = 3128, n total = 5766)							Cardiovascular mortality (n events = 621, n total = 5766)							Smoking-related mortality (n events = 571, n total = 5766)						
BW	n events / n total	HR			95% CI		p-value	n events / n total		HR		95% CI		p-value	n events / n total		HR		95% CI		p-value
< 2500	212/298	**1.71**			(1.45; 2.00)		**6.84e-11**	51/298		**2.16**		(1.54; 3.03)		**7.55e-06**	55/298		**2.49**		(1.78; 3.48)		**9.32e-08**
2500–2999	563/965	**1.23**			(1.20; 1.38)		**0.0004**	117/965		**1.41**		(1.09; 1.81)		**0.0081**	128/965		**1.65**		(1.24; 2.16)		**0.0002**
3000–3499	1236/2335	1.06			(0.97; 1.18)		0.1895	231/2335		1.05		(0.85; 1.29)		0.6703	233/2335		1.24		(0.99; 1.57)		0.0635
3500–3999	829/1637	1.00						171/1637		1.00					121/1637		1.00				
> 3999	288/531	1.06			(0.93; 1.23)		0.3794	51/531		0.87		(0.62; 1.20)		0.3913	34/531		0.97		(0.65; 1.46)		0.8942
Population of women who smoked during pregnancy^ǂ^																					
	All-cause mortality (n events = 1911, n total = 2989)							Cardiovascular mortality (n events = 357, n total = 2989)							Smoking-related mortality (n events = 475, n total = 2989)						
BW	n events / n total		HR	95% CI		p-value		n events / n total	HR		95% CI		p-value		n events / n total	HR		95% CI		p-value	
< 2500	169/220		**1.73**	(1.43; 2.09)		**1.37e-08**		36/220	**1.96**		(1.29; 2.97)		**0.0015**		53/220	**2.61**		(1.83; 3.74)		**1.41e-07**	
2500–2999	417/623		**1.32**	(1.14; 1.53)		**0.0001**		79/623	**1.42**		(1.02; 1.97)		**0.0372**		113/623	**1.60**		(1.19; 2.15)		**0.0018**	
3000–3499	789/1252		1.14	(1.01; 1.29)		**0.0406**		142/1252	1.07		(0.81; 1.42)		0.6373		197/1252	**1.35**		(1.04; 1.75)		**0.0244**	
3500–3999	432/732		1.00					87/732	1.00						89/732	1.00					
> 3999	104/162		0.94	(0.75; 1.18)		0.5991		13/162	**0.47**		(0.24; 0.90)		**0.0230**		23/162	1.01		(0.63; 1.64)		0.9562	
Population of women who did not smoke during pregnancy																					
	All-cause mortality (n events = 1217, n total = 2777)							Cardiovascular mortality (n events = 264, n total = 2777)							Smoking-related mortality (n events = 96, n total = 2777)						
BW	n events / n total		HR	95% CI		p-value		n events / n total	HR		95% CI		p-value		n events / n total	HR		95% CI		p-value	
< 2500	43/78		**1.51**	(1.08; 2.10)		**0.0160**		15/78	**2.33**		(1.28; 4.23)		**0.0055**		2/78	1.07		(0.25; 4.55)		0.9230	
2500–2999	146/342		0.91	(0.74; 1.12)		0.3642		38/342	1.15		(0.77; 1.74)		0.4972		15/342	1.33		(0.68; 2.62)		0.4058	
3000–3499	447/1083		0.97	(0.84; 1.12)		0.7042		89/1083	0.96		(0.70; 1.32)		0.7859		36/1083	1.06		(0.63; 1.77)		0.8374	
3500–3999	397/905		1.00					84/905	1.00						32/905	1.00					
> 3999	184/369		1.14	(0.95; 1.36)		0.1689		38/369	1.12		(0.75; 1.66)		0.5749		11/369	0.83		(0.39; 1.76)		0.6205	

Cox proportional hazard model adjusted for maternal age at birth, pre-pregnancy BMI, sex of offspring, gestational age at birth, marital and employment status, hypertension during pregnancy

*Adjusted for smoking in pregnancy

ǂAdjusted for cigarette consumption per day during pregnancy

*BW* Offspring birth weight category (grams), *HR* Hazard ratio, 95% CI = 95% Confidence Interval. *CVD* Cardiovascular disease. Cardiovascular mortality includes hypertension, ischemic heart disease, other heart diseases, diseases of the brain vessels, diseases in arteries, arterioles and capillaries, and other vascular diseases. Smoking-related mortality includes cancers in the oral cavity, oesophagus and airways, and chronic obstructive lung disease, asthma and bronchitis. Significant estimates are marked with bold.

In the population of women who smoked during pregnancy, cardiovascular mortality was higher in the groups of women giving birth to offspring with a BW < 2500 g (HR 1.96 [1.29–2.97]) or between 2500–2999 g HR 1.42 [95% CI 1.02–1.97]) and lower in the group of women giving birth to offspring above 3999 g (HR 0.47 [95% CI 0.24–0.90]) (Table 2). Estimates for all-cause and smoking-related mortality mirrored those in the total population (Table 2).

In the population of women who did not smoke during pregnancy, we observed a higher all-cause mortality and cardiovascular mortality in women giving birth to offspring with a BW below 2500 g compared with women giving birth to offspring with a BW within the reference category (all-cause mortality: HR 1.51 [95%CI 1.08–2.10], cardiovascular mortality: HR 2.33 [95%CI 1.28–4.23]) (Table 2). We did not observe lower all-cause or cardiovascular mortality in the group of women giving birth to offspring with a BW above 3999 g. There were no associations between offspring BW and smoking-related mortality in this population (Table 2).

### Morbidity

In the total populations, the hazards of MACE and stroke were higher in women giving birth to offspring with a BW below 2500 g (MACE: HR 1.35 [95% CI 1.01–1.79], stroke: HR 1.51 [95% CI 1.09–2.08]) and lower in women giving birth to offspring with a BW above 3999 g (MACE: HR 0.78 [95% CI 0.61–0.99], stroke: HR 0.78 [95% CI 0.59–1.03]) compared with the group of women giving birth to offspring with a BW within the reference category (3500–3999 g) (Table 3). Compared to women giving birth to offspring with a BW within the reference category, women giving birth to offspring with a BW above 3999 g had a lower hazard for hypertension (HR 0.82 [95% CI 0.68–0.99]).

**Table 3:** Cardiovascular morbidity in women in relation to offspring birth weight

	Total population of women* (n total = 5688)					Women who smoked during pregnancy^ǂ^ (n total = 2935)					Women who did not smoke during pregnancy (n total = 2753)				
BW	Diagnosis	n events / n total	HR	95% CI	p-value	Diagnosis	n events / n total	HR	95% CI	p-value	Diagnosis	n events / n total	HR	95% CI	p-value
< 2500	MACE (n = 1139)	61/291	**1.35**	(1.01; 1.79)	**0.0430**	MACE (n = 610)	43/214	1.39	(0.97; 1.99)	0.0690	MACE (n = 529)	18/77	1.37	(0.83; 2.27)	0.2157
2500–2999		180/949	0.97	(0.80; 1.17)	0.7324		114/609	1.04	(0.80; 1.35)	0.7611		66/340	0.86	(0.64; 1.16)	0.3250
3000–3499		470/2302	1.02	(0.88; 1.18)	0.8196		277/1230	1.20	(0.97; 1.47)	0.0883		193/1072	0.84	(0.68; 1.04)	0.1032
3500–3999		335/1620	1.00				151/722	1.00				184/898	1.00		
> 3999		93/526	**0.78**	(0.61; 0.99)	**0.0423**		25/160	**0.55**	(0.35; 0.88)	**0.0118**		68/366	0.89	(0.66; 1.19)	0.4199
< 2500	IHD (n = 957)	37/291	0.95	(0.66; 1.35)	0.7648	IHD (n = 500)	25/214	0.91	(0.58; 1.42)	0.5157	IHD (n = 457)	12/77	1.02	(0.55; 1.90)	0.9386
2500–2999		163/949	1.06	(0.86; 1.30)	0.5767		91/609	0.95	(0.71; 1.26)	0.6852		72/340	1.15	(0.85; 1.55)	0.3604
3000–3499		382/2302	0.97	(0.83; 1.14)	0.7449		217/1230	1.09	(0.87; 1.37)	0.1149		165/1072	0.85	(0.68; 1.08)	0.1830
3500–3999		284/1620	1.00				131/722	1.00				153/898	1.00		
> 3999		91/526	0.97	(0.75; 1.24)	0.7953		36/160	1.12	(0.75; 1.66)	0.9829		55/366	0.89	(0.65; 1.23)	0.4810
< 2500	Stroke (n = 842)	49/291	**1.51**	**(1.09; 2.08)**	**0.0125**	Stroke (n = 435)	35/214	**1.68**	(1.13; 2.51)	**0.0111**	Stroke (n = 407)	14/77	1.34	(0.76; 2.38)	0.3130
2500–2999		134/949	1.01	(0.81; 1.27)	0.9094		87/609	1.20	(0.88; 1.62)	0.2436		47/340	0.82	(0.58; 1.15)	0.2520
3000–3499		340/2302	1.01	(0.85; 1.19)	0.9323		191/1230	1.17	(0.92; 1.50)	0.2085		149/1072	0.85	(0.67; 1.09)	0.1970
3500–3999		250/1620	1.00				105/722	1.00				145/898	1.00		
> 3999		69/526	0.78	(0.59; 1.03)	0.0776		17/160	**0.56**	(0.33; 0.97)	**0.0396**		52/366	0.87	(0.63; 1.22)	0.420
< 2500	Hyper- tension (n = 1714)	66/291	0.99	(0.76; 1.28)	0.9229	Hyper-tension (n = 774)	41/214	0.98	(0.69; 1.37)	0.8908	Hyper- tension (n = 940)	25/77	1.00	(0.65; 1.51)	0.9811
2500–2999		270/949	1.04	(0.89; 1.21)	0.6202		154/609	1.09	(0.87; 1.36)	0.4441		116/340	0.94	(0.75; 1.18)	0.5985
3000–3499		699/2302	0.96	(0.85; 1.08)	0.4518		327/1230	0.98	(0.81; 1.17)	0.7916		372/1072	0.94	(0.80; 1.10)	0.4575
3500–3999		525/1620	1.00				297/722	1.00				318/898	1.00		
> 3999		154/526	**0.82**	(0.68; 0.99)	**0.0384**		45/160	0.91	(0.65; 1.28)	0.5974		109/366	**0.77**	(0.61; 0.98)	**0.02995**

COX proportional hazard model adjusted for maternal age at birth, pre-pregnancy BMI, sex of offspring, marital and employment status, and hypertension during pregnancy

*Adjusted for smoking in pregnancy

ǂAdjusted for cigarette consumption per day during pregnancy

*BW* Offspring birth weight category (grams), *HR* Hazard ratio, 95% CI = 95%. Confidence Interval. *MACE* Major cardiovascular events (acute myocardial infarction, stroke, cardiac arrest or sudden cardiac death, *IHD* Ischemic heart disease. Significant estimates are marked in bold.

After stratification into two populations by smoking status during pregnancy, the overall pattern of associations remained. However, slight differences were observed. In the population of women who smoked during pregnancy, the hazard for MACE in women giving birth to offspring below 2500 g was now borderline significant (HR 1.39 [95% CI 0.97–1.99]) but the hazards for stroke in the group of women giving birth to offspring below 2500 g or above 3999 g remained significant (HR 1.68 [95% CI 1.13–2.51], HR 0.56 [95% CI 0.33–0.97], respectively) (Table 3). The association between offspring BW and hypertension was not observed in this population (HR 0.91 [95% CI 0.65–1.28]).

In the population of women who did not smoke during pregnancy, a higher risk of MACE and stroke was observed for the group of women who gave birth to offspring with a BW below 2500 g compared with the reference, although estimates no longer reached statistical significance (MACE: HR 1.37 [95% CI 0.83–2.27], stroke: HR 1.34 [95% CI 0.76–2.38]) (Table 3). A significantly lower risk of hypertension in women giving birth to offspring above 3999 g compared with the reference (HR 0.77 [95%CI 0.61–0.98]) reappeared in this population (Table 3).

For IHD, no significant associations or patterns of associations with offspring BW were observed for any of the populations.

## Discussion

In this study, we aimed to assess the relation between delivery of a small term born infant and long-term maternal cardiovascular mortality and morbidity. We included women with previous singleton pregnancies, that were not complicated by diabetes, preeclampsia or preterm birth, adjusted our analyses for key confounding factors, and found that offspring BW was strongly inversely associated with long-term all-cause and cardiovascular mortality as well as cardiovascular morbidity. When we assessed women smoking and not smoking during pregnancy separately, the associations remained.

### Future maternal health after giving birth to a small baby

Women who had given birth to an infant with a BW below 3000 g had a higher all-cause and cardiovascular mortality compared with women who had given birth to infants within the reference BW category. The highest estimates were observed in women giving birth to babies below 2500 g, who had a 71% higher all-cause mortality and a 116% higher cardiovascular mortality. This inverse association is consistent with previous findings in women giving birth to small babies [6, 9, 23, 29], and suggests that giving birth to a small baby may reveal an underlying maternal vulnerability to developing CVD, thus offering a window of opportunity for timely intervention [4, 5]. Our analyses of cardiovascular morbidity confirm this potential underlying vulnerability as we observed a 35% higher risk of MACE and 51% higher risk of stroke in women giving birth to offspring with a BW below 2500 g compared to the reference. Previous studies have found strong inverse associations between offspring BW and subsequent risk of IHD in the mother [29–31], however, we did not replicate this finding in any of the populations studied, nor did we observe any consistent pattern of association, as all risk estimates were close to one. The exclusion of women with pre-eclampsia and preterm delivery from the study population may explain the absence of associations with IHD, given that both conditions are established risk factors for low offspring BW and subsequent maternal IHD [32, 33]. Our findings suggest that the higher risk of maternal IHD observed in previous studies, may be mainly due to confounding effects of other pregnancy complications.

A low offspring BW, if not accompanied by other pregnancy complications such as preeclampsia or preterm birth, is mainly caused by placental insufficiency [34]. A dysfunctional placenta could reflect underlying subclinical maternal cardiovascular impairment or have lasting programming effects on the maternal organs and physiology – either of which would increase the risk of maternal disease long-term. However, a large proportion of fetal growth restriction cases involve placentas with no signs of insufficiency, which may suggest that the baby has reached its genetically determined growth potential and is therefore constitutionally small rather than truly growth restricted [34]. Yet, delivery of a small infant may still reflect increased maternal disease risk, as genetic variants associated with elevated cardiovascular risk could restrict fetal growth when inherited by the fetus [35, 36]. Unfortunately, we were unable to explore this distinction, as neither genetic information nor placental weight, function, or pathology data were available in this study.

### Future maternal health after giving birth to a large baby

Giving birth to an infant above 3999 g was found to be associated with lower cardiovascular mortality (16% lower in the total population and 54% lower in the population of women who smoked during pregnancy) compared to the reference. In addition, we found a 22% lower risk of MACE and stroke, and 18% lower risk of hypertension in women giving birth to offspring above 3999 g. These findings contrast with most previous studies, which have reported higher mortality in women giving birth to large babies [37, 38]. We excluded women with diabetes, which is also known to be increasing offspring BW [39] and subsequent maternal cardiovascular risk [18, 40], from our study population. Moreover, a previous study reported that a high offspring BW was associated with a higher maternal cardiovascular mortality, if the baby was born prematurely but that the association was absent if the baby was born at term [7]. This finding, together with the results of the present study, may imply that women who give birth to large babies may have a lower risk of CVD – provided the pregnancy was not complicated by hyperglycemia or preterm delivery.

### Stratification by smoking status in pregnancy

A key finding of this study was that the pattern of an inverse associations between offspring BW and long-term maternal morbidity and mortality remained after dividing the population according to smoking status in pregnancy, reflecting that we found no significant interaction between smoking during pregnancy and offspring BW. Moreover, associations between offspring BW and maternal cardiovascular outcomes were present in both populations despite the lower power in the population of women who did not smoke during pregnancy – only 44% were deceased at follow-up vs. 64% deceased at follow-up in the population of women who smoked during pregnancy.

Giving birth to a small infant (< 2500 g) was strongly associated with higher all-cause and cardiovascular mortality both among women who smoked during pregnancy and those who did not. Although the association between offspring BW < 2500 g and maternal risk of MACE was no longer significant, the pattern of higher maternal cardiovascular morbidity among women who gave birth to infants with a BW < 2500 g – compared to the reference group – persisted after stratification by smoking status during pregnancy. These and previous findings suggest that associations between offspring BW and future maternal health, are not solely attributable to the adverse effects of smoking during pregnancy. Moreover, the similar pattern of associations across strata indicates that fetal growth restriction may reflect future maternal cardiovascular health regardless of smoking behavior during pregnancy. Smoking during pregnancy is known to cause placental insufficiency [41, 42]. Accordingly, placental insufficiency in pregnancies affected by maternal smoking may either reflect an increased sensitivity towards the adverse cardiovascular effects of smoking, and/or placental dysfunction may program maternal biology towards an elevated risk of CVD [43].

The lower risk of MACE and stroke in women who delivered infants with a BW above 3999 g was most pronounced among those who smoked during pregnancy, whereas the strongest association with reduced risk of hypertension was observed among non-smoking women who delivered infants with a BW above 3999 g. Although differences in the underlying mechanisms between the two populations cannot be ruled out, these findings may be explained by limited statistical power.

A markedly lower number of women with hypertension during pregnancy was observed in the women who smoked during pregnancy (15% vs. 25%, Supplementary table S2), which is in line with the previously shown inverse association between smoking during pregnancy and hypertensive disorders in pregnancy [44].

To meet the needs of the developing fetus, maternal physiology undergoes substantial adaptations throughout pregnancy [45]. If maternal physiology fails to meet the demands of pregnancy, this may lead to complications such as fetal growth restriction, which could reflect limited functional capacity and thereby underlying cardiovascular impairment [46]. A pregnancy complicated by fetal growth restriction may also have programming effects on maternal physiology thereby increasing the risk of subsequent CVD. Regardless of the mechanisms behind, our findings add to the evidence that offspring BW may serve as an independent marker of maternal long-term health and disease and not merely reflect effects of potential confounding factors.

### Strengths and limitations

A strength of this study is the level of information about the women before and during pregnancy – especially the detailed information about maternal smoking during pregnancy. This information, along with information on smoking-related causes of death, made it possible for us to separate the effects of smoking from the effects of other factors affecting fetal growth on long-term maternal health and disease. Our analyses are also strengthened by the prospective design of the study, which minimizes recall bias, and by the long follow-up period of up to 45 years. Further, the validity and coverage of the register data [26, 27] is a strength of this study.

This study also has several limitations. A major limitation is that we did not have information on the woman’s own BW, which would have been relevant to account for in the analyses as own BW has a significant impact on long-term health and disease and is strongly correlated with offspring BW [47]. Employment status in pregnancy was assessed as yes/no but it is unclear what employment during pregnancy indicated in Denmark around the year of 1960, as employment – and when in pregnancy the woman was employed – could indicate both high and low social class (e.g. could indicate both financial security and social vulnerability if employment indicated that the women was the only provider). We attempted to address this by adjusting for both employment and marital status. However, residual confounding by socioeconomic status (SES) may persist, as SES is likely associated with both the exposure (offspring BW) and the outcomes (cardiovascular morbidity and mortality).

The exclusion criteria applied to minimize confounding in this study reduced the statistical power of our analyses, which may have weakened observed associations or prevented detection of additional associations. The exclusion of women, who were deceased before follow-up start, could also have affected the outcomes, especially if there was a different distribution of offspring BW in the groups excluded.

## Conclusion

Our findings reveal a higher cardiovascular mortality and risk of MACE and stroke in women with pregnancies involving fetal growth restriction, but not complicated by preterm delivery, pre-eclampsia, or diabetes. Our findings of lower cardiovascular morbidity and mortality among women who gave birth to large babies, along with the absence of an association between offspring BW and maternal IHD, underscore the importance of accounting for confounding factors when studying such associations. Furthermore, we show that these associations are present regardless of smoking behavior during pregnancy. This highlights that, irrespective of the underlying cause of fetal growth restriction, the future health of mothers who gave birth to growth restricted babies should be carefully considered to improve long-term health, promote longevity, and prevent CVD.

Future studies should investigate the role of the placenta and the shared genetic architecture of fetal growth restriction and maternal CVD to elucidate mechanisms underlying the associations and to assess potential causality.

## Supplementary Information

Below is the link to the electronic supplementary material.

Supplementary file1 (PDF 1075 KB)

## Abbreviations

BW: Birth weight
GA: Gestational age
CVD: Cardiovascular disease
DCDR: Danish cause of death register
DNPR: Danish national patient register
CPR: Central person register
ICD: International classification of disease
MACE: Major cardiovascular events
IHD: Ischemic heart disease
HR: Hazard ratio
CI: Confidence interval
