# Supplementary material for: Birth weight of term born offspring in relation to long-term maternal cardiovascular morbidity and mortality
Source: Eur J Epidemiol. 2026 Feb 21;41(5):587–98. doi: 10.1007/s10654-025-01355-1 (PMC13332883; doi:10.1007/s10654-025-01355-1)
Supplement: Supplementary file 1 — Supplementary file1 (PDF 1075 KB) [file 10654_2025_1355_MOESM1_ESM.pdf]

Supplementary Information (SI) for:

Birth Weight of Term Born Offspring in relation to Long-Term Maternal Cardiovascular Morbidity and Mortality

Pauline Kromann Reim (ORCID-ID: 0009-0002-0135-8218)[1,2], Line Engelbrechtsen (ORCID-ID: 0000-0002-0666-0830)[1,2,7], Lise Geisler Bjerregaard (ORCID-ID: 0000-0001-8471-0832)[3], Louise Kelstrup (ORCID-ID: 0000-0003-3994-0377)[2,7], Erik Lykke Mortensen (ORCID-ID: 0000-0002-6985-451X)[4,5], Thorkild IA Sørensen (ORCID-ID: 0000-0003-4821-430X)[1,4,6], Torben Hansen (ORCID-ID: 0000-0001-8748-3831)[1*]

[1]Novo Nordisk Foundation Center for Basic Metabolic Research, University of Copenhagen, Denmark

[2]Department of Gynecology and Obstetrics, Herlev University Hospital, Denmark

[3]Center for Clinical Research and Prevention, Copenhagen University Hospital – Bispebjerg and Frederiksberg, Copenhagen, Denmark

[4]Department of Public Health, University of Copenhagen, Copenhagen, Denmark

[5]Center for Healthy Aging, University of Copenhagen, Copenhagen, Denmark

[6] Center for Childhood Health, Copenhagen, Copenhagen, Denmark

[7]Dept of Clinical Medicine, University of Copenhagen, Copenhagen, Denmark

*Corresponding author: Torben Hansen (torben.hansen@sund.ku.dk)

Submitted to the European Journal of Epidemiology

Fig. S1a Model check of linearity in the association between offspring BW and maternal all-cause mortality in the total population

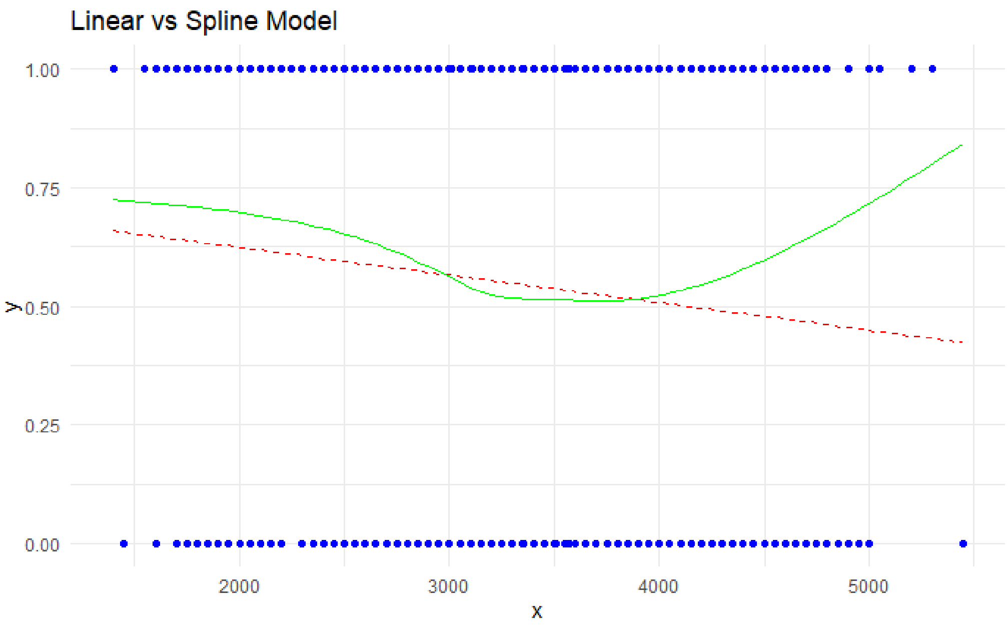

The dotted red line is the linear model, and the light green is the spline model. X-axis represents birth weight (g) and y-axis is the status at end of follow-up (1 = dead, 0 = alive).
Anova analyses revealed that the non-linear model was superior to the linear model (p = <0.001)

Fig. S1b Model check linearity in the association between offspring BW and maternal all-cause mortality in a population without offspring birth weight outliers (defined as <1500 grams or >4500 grams)

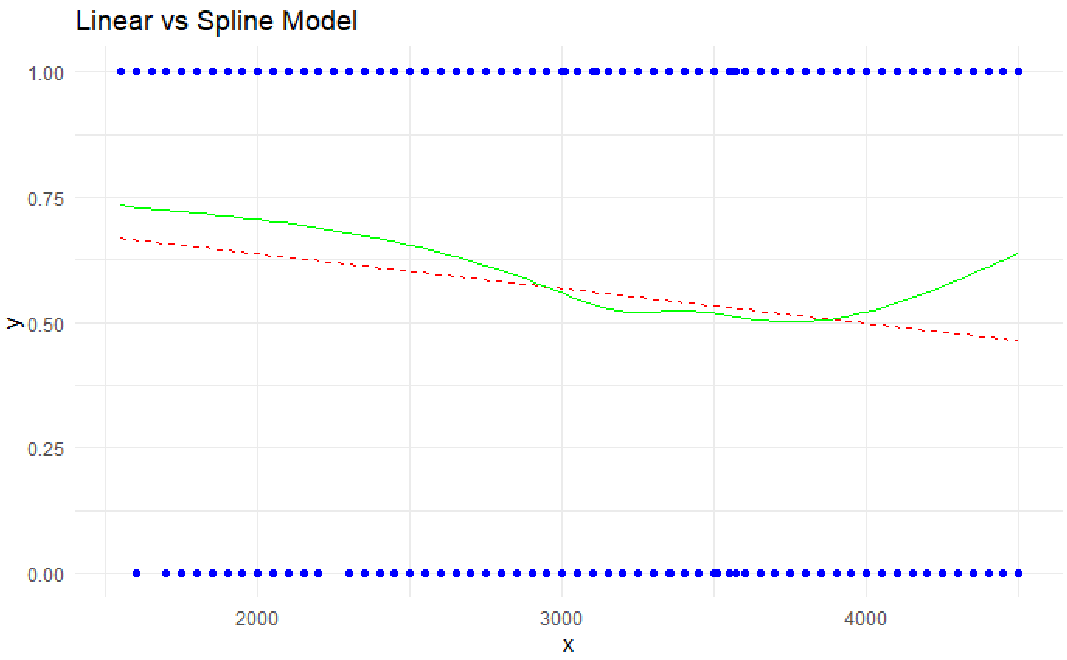

The dotted red line is the linear model, and the light green is the spline model. X-axis represents birth weight (g) and y-axis is the status at end of follow-up (1 = dead, 0 = alive).

Table S1: Population Characteristics stratified by Smoking Status in Pregnancy

**Smoking during pregnancy**	**No**	**Yes**
n	2777	2989
Offspring Birth Weight (g) (mean (SD))	3430 (487)	3220 (477)
Offspring Birth Length (cm) (mean (SD))	52 (2)	51 (2)
Gestational Age at Delivery (weeks) (mean (SD))	40.4 (1.78)	40.5 (1.96)
Sex of Offspring = M (%)	1425 (51.3)	1483 (49.6)
Caesarean Section = Yes (%)	191 (6.9)	179 (6.0)
Maternal Age (years) (mean (SD))	26 (7)	25 (6)
Pre-pregnancy BMI (mean (SD))	22.0 (2.89)	21.5 (2.75)
Marital Status = Not married (%)	959 (34.5)	1313 (43.9)
Employed in Pregnancy = Yes (%)	1746 (62.9)	2007 (67.1)
Hypertension in Pregnancy = Yes (%)	680 (24.7)	435 (14.7)

Fig. S2 Offspring birth weight according to daily cigarette consumption in pregnancy

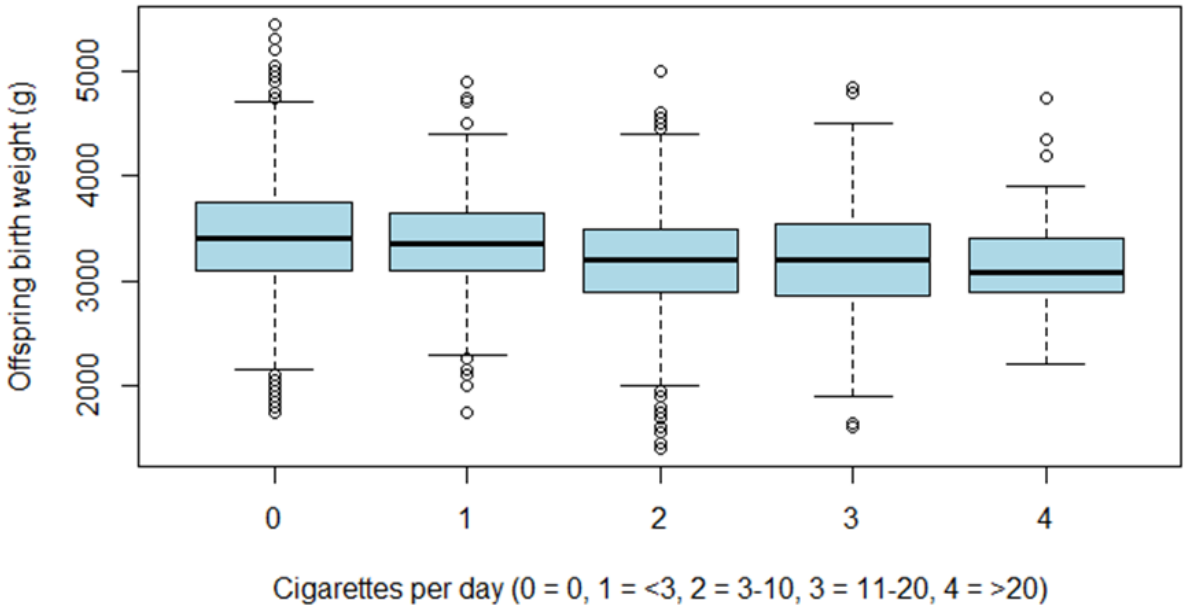

Number of women in each group: 0 = 2826, 1 = 440, 2 = 1742, 3 = 717, 4 = 86, NA = 70. Total = 5881.
Offspring BW was significantly lower in women who smoked (any number of cigarettes) during pregnancy compared with offspring BW in women who did not smoke in pregnancy.

Table S2: Difference in birth weight (g) according to cigarettes smoked per day in pregnancy

Comparison of categories of cigarettes consumption per day in pregnancy	Difference in birth weight (g) (95%CI)	P-value*
<3 vs. 0	-85.51 (-152.66; -18.37)	0.005
3-10 vs 0	-223.82 (-263.73; -183.91)	< 0.001
11-20 vs. 0	-251.09 (-305.88; -196.31)	< 0.001
>20 vs. 0	-294.77 (-438.17; -151.36)	< 0.001
3-10 vs. <3	-138.31 (-208.21; -68.41)	< 0.001
11-20 vs. <3	-165.58 (-244.92; -86.24)	< 0.001
>20 vs. <3	-209.25 (-363.72; -54.79)	0.002
11-20 vs. 3-10	-27.27 (-85.40; 30.86)	0.703
>20 vs. 3-10	-70.94 (-215.66; 73.78)	0.668
>20 vs. 11-20	-43.67 (-193.18; 105.83)	0.932

Number of women in each group: 0 = 2826, 1 = 440, 2 = 1742, 3 = 717, 4 = 86, NA = 70. Total = 5881.
*Adjusted for multiple testing.

Table S3: Schoenfeld Residuals for covariates included in the cox-regression model

Schoenfeld Residuals			
	Chisq	Degrees of freedom	p-value
BW category	8.10308	4	0.23276
Smoking	7.91118	1	**0.00044**
Maternal age at birth	3.00267	1	**0.00016**
Maternal pre-prenancy BMI	7.96397	1	**0.00004**
Offspring sex	0.01047	1	0.90483
Gestational age at birth	0.00744	1	0.70578
Marital status	3.08690	1	0.41026
Employment	0.03587	1	0.17497
Hypertension	0.05475	1	0.48493
GLOBAL	34.04443	12	2.4e-08

Fig. S3a Schoenfeld Residual Plot illustrating the effect of smoking status in pregnancy on maternal mortality with increasing age

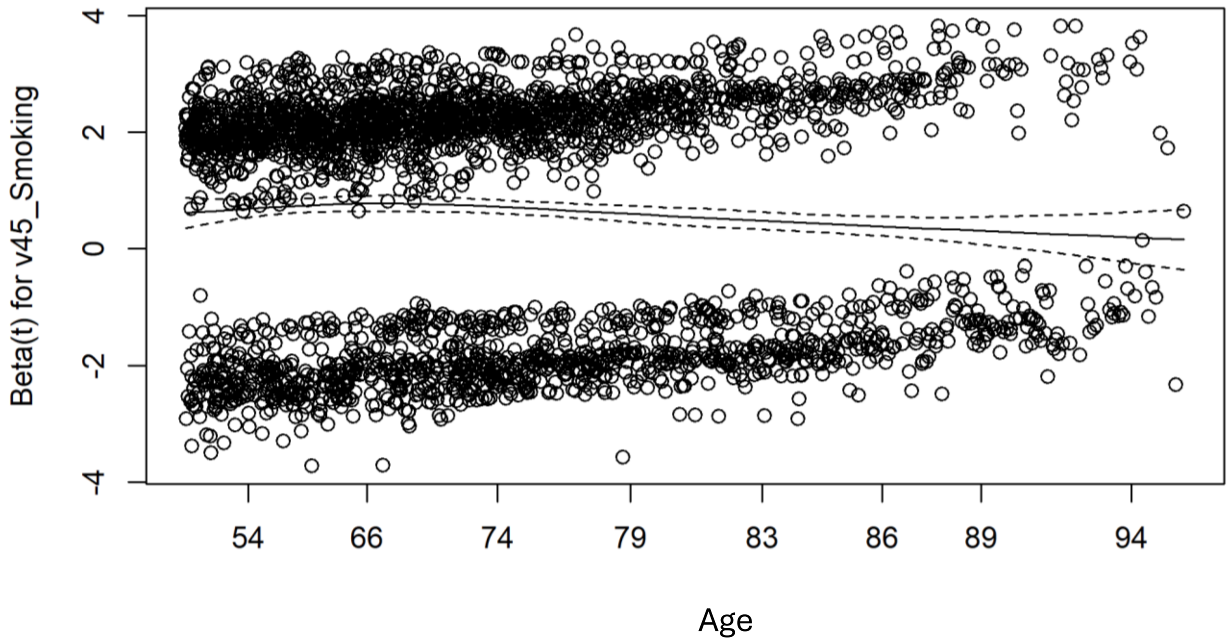

Fig. S3b Schoenfeld Residual Plot illustrating the effect of maternal pre-pregnancy BMI on maternal mortality with increasing age

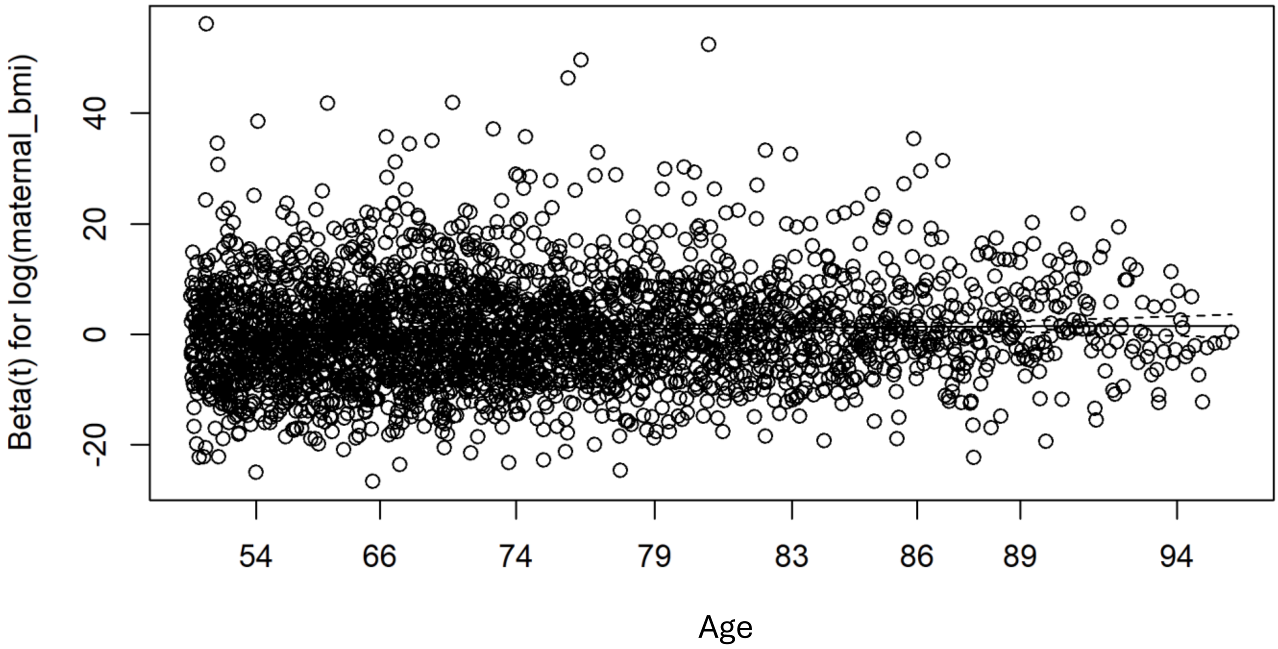

Fig. S3c Schoenfeld Residual Plot illustrating the effect of maternal age at birth on maternal mortality with increasing age

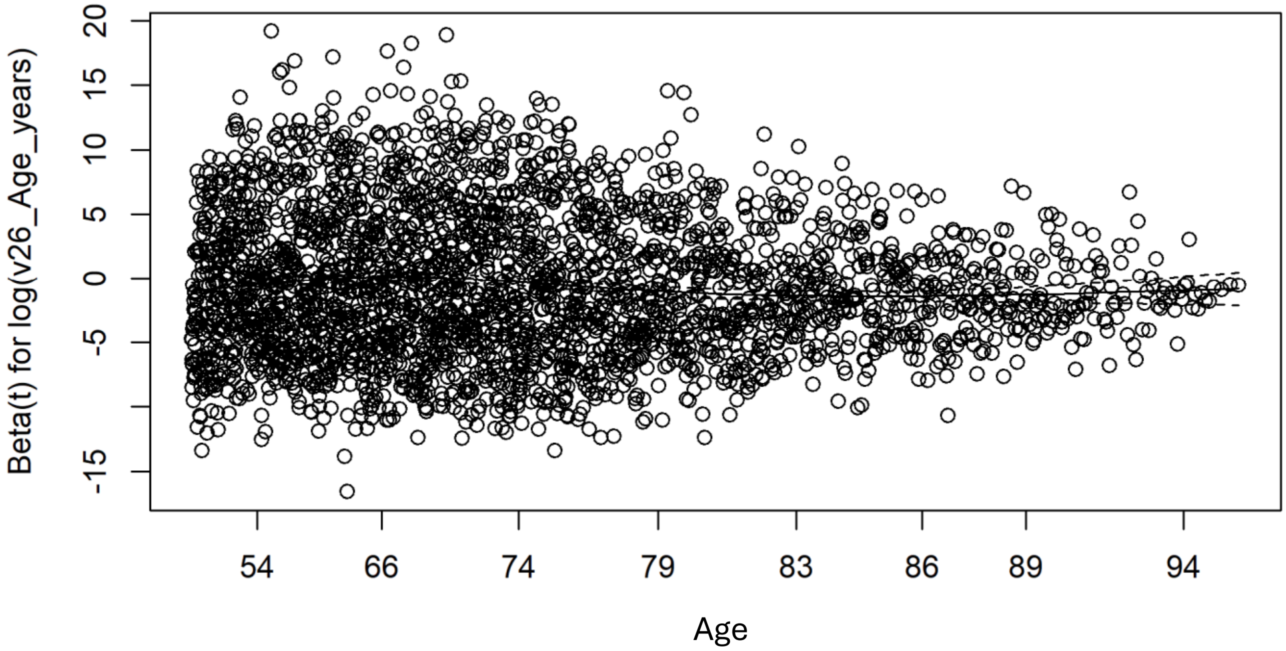

Table S4a: Likelihood ratio test analysis of smoking as a confounder (reduced model = without smoking as a covariate, full model = with smoking as a covariate)

	Loglik	Chisq	Df	P-value*
All-cause mortality	-22990	226.42	1	< 2.2e-16
Cardiovascular mortality	-4529.9	31.854	1	1.662e-08
Smoking-related mortality	-4174.0	257.29	1	< 2.2e-16
MACE	-8641.9	25.862	1	3.667e-07
IHD	-5624.5	18.928	1	1.357e-05
Stroke	-6425.9	10.781	1	0.0010
Hypertension	12908	0.456	1	0.4995

*P-value <0.05 indicates that the full model is superior to the reduced model.

Table S4b: Likelihood ratio test analysis of smoking as an effect modifier (reduced model = with smoking as a covariate, full model = with smoking as a covariate and with interaction term between smoking and birth weight)

	Loglik	Chisq	Df	P-value*
All-cause mortality	-22986	8.1005	4	0.0880
Cardiovascular mortality	-4525.3	9.1998	4	0.0563
Smoking-related mortality	-4172.9	2.2253	4	0.6944
MACE	-8636.6	10.534	4	0.0323
IHD	-5621.4	6.1104	4	0.1911
Stroke	-6422.7	6.3227	4	0.1763
Hypertension	-12907	1.1676	4	0.8834

*P-value <0.05 indicates that the full model is superior to the reduced model.

Fig. S4 Crude survival according to offspring BW in all women

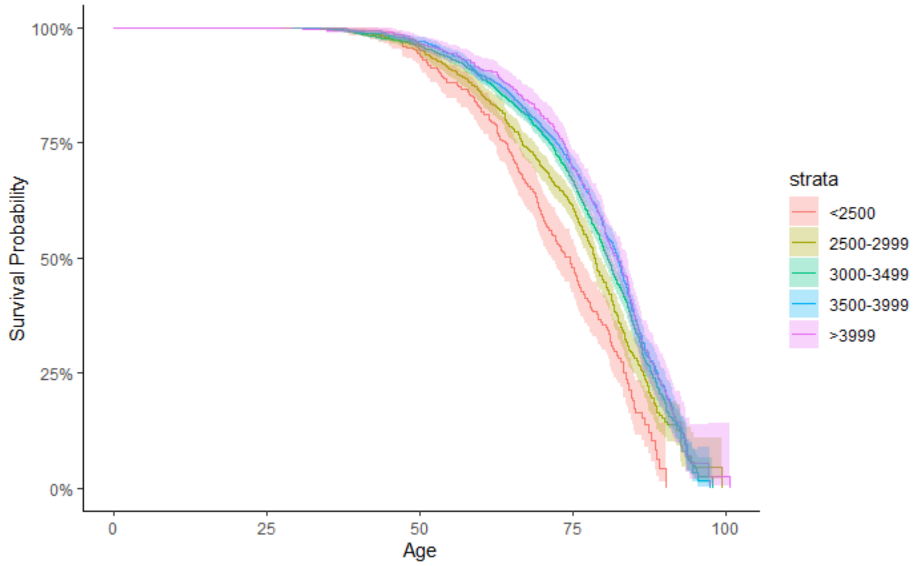

Survival curves of all women during pregnancy according to offspring BW category. Analyses are crude and not adjusted for any covariates.

**Pairwise comparison using Log-Rank test (p-values)**				
	<2500	2500-2999	3000-3499	3500-3999
2500-2999	3.6e-05	.	.	.
3000-3499	1.9e-13	0.00028	.	.
3500-3999	< 2e-16	2.8e-07	0.02859	.
>3999	1.8e-13	3.6e-05	0.06937	0.73699

Fig. S5 Crude survival according to offspring BW in women who smoked during pregnancy

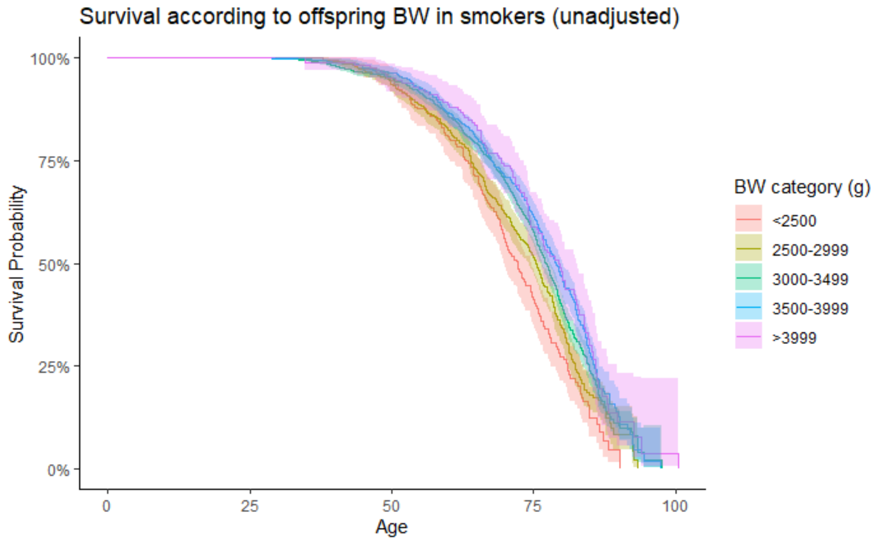

Survival curves according to offspring BW category of women who smoked during pregnancy. Analyses are crude and not adjusted for any covariates.

Pairwise comparison using Log-Rank test (p-values)				
	<2500	2500-2999	3000-3499	3500-3999
2500-2999	0.0260	.	.	.
3000-3499	9.7e-06	0.0041	.	.
3500-3999	4.7e-08	1.6e-05	0.0358	.
>3999	3.5e-05	0.0041	0.1392	0.7423

Fig. S6 Crude survival according to offspring BW in women who did not smoke during pregnancy

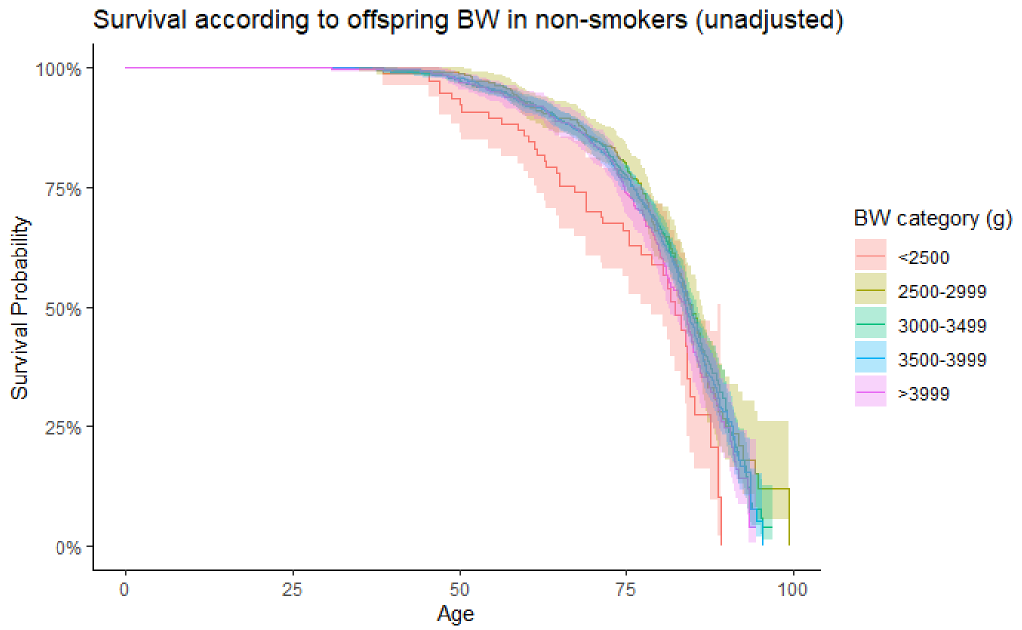

Survival curves according to offspring BW category of women who did not smoke during pregnancy. Analyses are crude and not adjusted for any covariates.

**Pairwise comparison using Log-Rank test (p-values)**				
	<2500	2500-2999	3000-3499	3500-3999
2500-2999	0.025	.	.	.
3000-3499	0.024	0.631	.	.
3500-3999	0.025	0.463	0.608	.
>3999	0.121	0.205	0.205	0.341

Table S5: Distribution of women in each offspring BW category divided in women who did and did not smoke during pregnancy

Offspring BW (g)	Non-smokers, n (%)	Smokers, n (%)
<2500	78 (2.8)	220 (7.4)
2500-2999	342 (12.3)	623 (20.8)
3000-3499	1083 (39.0)	1252 (41.9)
3500-3999	905 (32.6)	732 (24.5)
>3999	369 (13.3)	162 (5.4)
Total number	2777	2989

BW = birth weight. There is a higher percentage of low and very low offspring birth weight in the group of women who smoked during pregnancy. A correspondingly lower percentage of high offspring birth weight in the same group. This is reflected in the 200 grams lower average offspring birth weight in the group of women who smoked during pregnancy compared to women who did not (Table 2).
